# A stakeholder analysis to prepare for real-world evaluation of integrating artificial intelligent algorithms into breast screening (PREP-AIR study): a qualitative study using the WHO guide

**DOI:** 10.1186/s12913-024-10926-z

**Published:** 2024-05-02

**Authors:** Rumana Newlands, Hanne Bruhn, Magdalena Rzewuska Díaz, Gerald Lip, Lesley A. Anderson, Craig Ramsay

**Affiliations:** 1 Health Services Research Unit, University of Aberdeen, Aberdeen, UK; 2 North East Scotland Breast Screening Programme, NHS Grampian, Aberdeen, UK; 3 Centre for Health Data Science, Institute of Applied Health Sciences, University of Aberdeen, Aberdeen, UK

**Keywords:** Artificial intelligence, Algorithm, Breast, Screening, Stakeholder analysis

## Abstract

**Background:**

The national breast screening programme in the United Kingdom is under pressure due to workforce shortages and having been paused during the COVID-19 pandemic. Artificial intelligence has the potential to transform how healthcare is delivered by improving care processes and patient outcomes. Research on the clinical and organisational benefits of artificial intelligence is still at an early stage, and numerous concerns have been raised around its implications, including patient safety, acceptance, and accountability for decisions. Reforming the breast screening programme to include artificial intelligence is a complex endeavour because numerous stakeholders influence it. Therefore, a stakeholder analysis was conducted to identify relevant stakeholders, explore their views on the proposed reform (i.e., integrating artificial intelligence algorithms into the Scottish National Breast Screening Service for breast cancer detection) and develop strategies for managing ‘important’ stakeholders.

**Methods:**

A qualitative study (i.e., focus groups and interviews, March-November 2021) was conducted using the stakeholder analysis guide provided by the World Health Organisation and involving three Scottish health boards: NHS Greater Glasgow & Clyde, NHS Grampian and NHS Lothian. The objectives included: (A) Identify possible stakeholders (B) Explore stakeholders’ perspectives and describe their characteristics (C) Prioritise stakeholders in terms of importance and (D) Develop strategies to manage ‘important’ stakeholders. Seven stakeholder characteristics were assessed: their knowledge of the targeted reform, position, interest, alliances, resources, power and leadership.

**Results:**

Thirty-two participants took part from 14 (out of 17 identified) sub-groups of stakeholders. While they were generally supportive of using artificial intelligence in breast screening programmes, some concerns were raised. Stakeholder knowledge, influence and interests in the reform varied. Key advantages mentioned include service efficiency, quicker results and reduced work pressure. Disadvantages included overdiagnosis or misdiagnosis of cancer, inequalities in detection and the self-learning capacity of the algorithms. Five strategies (with considerations suggested by stakeholders) were developed to maintain and improve the support of ‘important’ stakeholders.

**Conclusions:**

Health services worldwide face similar challenges of workforce issues to provide patient care. The findings of this study will help others to learn from Scottish experiences and provide guidance to conduct similar studies targeting healthcare reform.

**Study registration:**

researchregistry6579, date of registration: 16/02/2021.

**Supplementary Information:**

The online version contains supplementary material available at 10.1186/s12913-024-10926-z.

## Introduction

The National Breast Screening Programme (NHSBSP) in the United Kingdom (UK) invites women aged 50–70 years every three years for X-ray mammography [1, 2]. The mammograms are digital images assessed independently by two readers (usually a radiologist and/or advanced radiography practitioner) for signs of cancer [3, 4]. Each reader decides whether the image appears normal or needs a recall for further assessment. In case of disagreement, arbitration involves a third reader [5, 6]. The NHSBSP detects between 17,000 and 20,000 breast cancers annually [3, 5]. Ongoing reader shortages may affect patient care by delaying breast cancer diagnosis and treatment [7]. With the NHSBSP being temporarily paused in 2020 because of the Covid-19 pandemic, the service is under additional pressure to deal with backlogs [8]. Evidence suggests that artificial intelligence (AI) can potentially transform healthcare delivery by improving care processes and patient outcomes [9–13]. Several studies have demonstrated positive outcomes from the application of AI in breast screening, particularly for image reading and triage [6, 13–15]. Research on the clinical and organisational benefits of AI is still at an early stage, with limited evidence to demonstrate how AI algorithms could benefit breast screening programmes [6, 12]. Numerous concerns have been raised around the possible implications of AI for patient safety, data security, public acceptance and trust, accountability for decisions and impact in the broader healthcare system [12, 16].

The Industrial Centre for Artificial Intelligence Research in Digital Diagnostics (iCAIRD, https://icaird.com/about/), a pan-Scotland multistakeholder programme, sought to evaluate an AI system targeting breast cancer detection in the real-world setting of the Scottish National Breast Screening Service. Modifying usual care through reform (e.g., integrating technologies to improve services) is a complex endeavour because the health care system is shaped and influenced by a diverse set of stakeholders [17, 18]. Stakeholder engagement at the early stages of system reform could help increase support for the proposed reform [18–20]. Stakeholder analysis is a practical tool to identify the key stakeholders (who may have a vested interest) in a reform process and develop strategies to engage with them early to mitigate issues targeting its successful implementation [17]. It can be defined as a process of systematically gathering and analysing qualitative information to determine whose interests should be considered when developing and/or implementing a policy or program [20]. As part of the iCAIRD programme, a stakeholder analysis was conducted to systematically identify stakeholders, explore their views around the reform (i.e., integrating AI algorithm into the Scottish National Breast Screening Service for breast cancer detection) and develop strategies for managing ‘important’ stakeholders [18, 20]. We defined ‘stakeholders’ as a person representing a group of individuals or an organisation who have a direct or indirect interest in, or the potential to influence or be affected by, the reform. This paper explains the detailed process and findings of our stakeholder analysis.

## Methods

The World Health Organisation (WHO) guide was followed for conducting the stakeholder analysis [20]. The objectives of the study included: (A) Identify possible stakeholders (B) Explore stakeholders’ perspectives and describe their characteristics (C) Prioritise stakeholders in terms of importance and (D) Develop strategies to manage ‘important’ stakeholders (see Additional file 1 for steps involved). Seven stakeholder characteristics were assessed based on the guide: their knowledge of the targeted reform, position, interest, alliances, resources, power and leadership (see Additional file 2 for description, rating and interpretation).

The study setting involved three Scottish health boards: NHS Greater Glasgow & Clyde, NHS Grampian and NHS Lothian. Due to the time of the study (the COVID pandemic) and limited resources and time, we approached these three health boards, which cover a wide geographical area and a large number of the Scottish population. These health boards were also involved in developing and training AI technologies and testing patient data. Ethical approval was received from the University of Aberdeen School of Medicine, Medical Sciences and Nutrition Ethics Review Board (Ref: CERB/2020/11/1997) and approved by each health board’s NHS Research and Development (R&D) office: NHS Grampian (Ref no: 282,136), NHS Greater Glasgow & Clyde Health Board (Ref no: GN20ON578) and NHS Lothian (Ref no: 2020/0234).

A list of possible stakeholders was generated (objective A) and finalised after discussion with the iCAIRD team. Three questions were considered during this task (as criteria to identify and select stakeholders): ‘*Who may affect or be affected by the reform?’*, *‘Who has the power to make/stop it from happening?’* and *‘Who are the potential allies/opponents of the targeted reform?’* The final list was grouped as: (a) women, (b) patient and public representatives (e.g., Scotland-based patient advocacy groups and cancer charities) and (c) professionals [e.g., clinical (e.g., readers), non-clinical staff (e.g., hospital board members, procurement), private companies (e.g., AI developer)]. Various subgroups or stakeholder types were then listed under these three main groups. Stakeholders were further categorised as internal (if working within the NHS organisation) or external (all other stakeholders). Stakeholders from the final list were then prioritised (i.e., most to least ranked based on their roles, experiences and positions per organisation and the three health boards) and contacted to explore their perspectives on the proposed reform (objective B).

Focus groups (with women) and semi-structured interviews (with professionals and patient and public representatives) were conducted (March-November 2021) via Microsoft Teams® or telephone. All data collection tools (provided by the WHO guide e.g., topic guide, ) were adapted, piloted and finalised to meet the study aim. Additional file 3 and 4 presents the final versions of the topic guides (targeting professionals, and for women and patient and public representatives) and Additional file 5 presents the vignettes (an additional tool developed). Additional questions (e.g., concerns, possible solutions, challenges towards or expectations following the reform), beyond the WHO guide, were added to capture a more comprehensive view of the proposed reform based on stakeholder role and the organisation they represented. Prioritised stakeholders (from objective A) were approached in March 2021 with a recruitment target of approximately 40 participants from the three groups. Professionals and patient and public representatives were invited via email by the clinical director (GL) of the North-East Scotland Breast Screening Programme, including an invitation letter and participant information leaflet. Radiology unit leads circulated the invitation among clinical staff across each health board. Various patient representative groups (e.g., cancer charities) were approached to recruit patient and public representatives. Women from the Aberdeen Children of the 1950s cohort study (aged 64–70 years) were invited through a Facebook page advert. The study advert was shared further in the social media by cohort members and hence, additional women outside the target group participated in the study. Additional participants, suggested by study interviewees, mainly in the professional group, were also approached. For stakeholders representing more than one NHS organisational role, their main role was considered at the interview and analysis. Only focus group participants and patient and public representatives were reimbursed for their time (£15 retail e-vouchers). Informed verbal consent (via TEAMS or telephone, by going through the consent form and asking to agree to each point) was obtained from each participant and recorded (using an audio recorder) before data collection, with sessions recorded and transcribed by a third party verbatim. All anonymised transcripts were imported into NVivo (version 12), content analysed by generating themes using the WHO guide [21]. For consistency, 10% of the transcripts (*n* = 3) were double-coded (by RN and HB, 98% concordance). All findings were presented (February 2022) to the iCAIRD team with the aim of finalising the ‘important’ stakeholders’ list (objective C). According to the WHO guide, stakeholders are ‘important’ if they have the power to influence the reform. In our study, we defined ‘important’ as stakeholders (no matter how much influence they have), whose wants, and needs should be considered, prioritised and addressed to maintain and/or increase support towards the proposed reform.

Finally, prototype strategies and their possible actions were generated targeting all or specific sub-groups of ‘important’ stakeholders. These strategies were finalised (objective D) along with considerations after discussion (via Microsoft TEAMS® by RN during April, 2022) with the iCAIRD and research teams. It should be noted that these strategies need to be co-produced (e.g., the contents, topics, when, how and by whom should be implemented) in further detail and validated involving targeted stakeholders before implementation (which was not part of this study) [22].

## Results

Seventeen stakeholder subgroups were included in the stakeholder list. Thirty-two participants, who took part in the study, represented 14 (out of seventeen) subgroups. Five stakeholder sub-groups were external, and the remainder were internal. See Table 1 for the types of stakeholders who took part in the study and findings of their characteristics. To preserve anonymity names, gender, positions, and health boards are not disclosed.

**Table 1:** Possible stakeholders’ groups, sub-groups and numbers that took part in the study and findings of their characteristics

Stakeholder group	Stakeholder types/sub-group	Description of roles in the context of the proposed reform	The number (Who took part in the study from three sites)	Internal or External (Works for NHS or not)	Influence (Based on power & leadership analysis)	Knowledge levels (3 = a lot, 2 = some, 1 = none)	^*^Position (S, MS, N, O, MO)	Alliances (Yes = willing to collaborate, No = not willing to collaborate)	Interests (Yes = interested and expressed advantages/disadvantages of the reform, No = not interested in the reform)
**Professionals**	1. Clinical directors of Scottish Breast Screening Programme	Lead the Breast Screening Programme at their catchment areas.	1	Internal	High potential	3	S	Yes	Yes
	2. Hospital management group	Approve new policies, provide access to patients’ data for AI’s evaluation, contribute to business cases, and address workforce issues.	4	Internal	High to moderate potential	3	S	Yes	Yes
	3. Scottish Breast Screening Programme Board members	Work towards evidence synthesis (e.g., AI’s performance in real-world settings) and feed information higher up through their channels for approvals.	1	Internal	High potential	3	S	Yes	Yes
	4. National Services Scotland (NSS)	Provide procurement, advice, logistics and distribution of services based on evidence.	1	Internal	High potential	3	MS	Yes	Yes
	5. National Screening Oversight Function Board (NSO) member	Provide oversight across all aspects of new and existing screening pathways within NHS Scotland.	1	Internal	High potential	3	S	Yes	Yes
	6. AI developing company	Develop and test AI systems and was working with NHS at the time of this study towards evidence synthesis.	1	External	High potential	3	S	Yes	Yes
	7. Radiologists	Read images and/or provide quality assurance services in mammography	1	Internal	Moderate potential	3	S	Yes	Yes
	8. National Services Division (NSD) member	Commission six screening centres and manage resources and solve technical and staff issues	2	Internal	Moderate potential	3	S	Yes	Yes
	9. Scottish AI Alliance	Deliver service on the vision for Scotland to become a leader in the development and use of trustworthy, ethical and inclusive AI. It is a partnership between the Data Lab and the Scottish government.	1	External	Moderate potential	3	S	Yes	Yes
	10. Health Technology review group	Provide advice on health technologies based on independent assessments of technologies’ performance and impact on the service.	1	External	Moderate potential	3	MS	Yes	Yes
	11. Radiographers	Help with taking mammography/images and some are qualified to read images	2	Internal	Low potential	3	MS or MO	Yes	Yes
	12. Innovation research group	Conduct research work in the area of technologies evaluation involving industry, academia and health and social care	1	Internal	Low potential	2	S	Yes	Yes
	13. UK National Screening Committee (UK NSC)	Approve a new policy for all four countries. The Scottish Govt follows their advice in deciding funding and approvals	Declined participation	Internal	N/A	N/A		N/A	N/A
	14. National (Scottish) Screening Council (NSC)	Oversee screening and recommend what tools should be used in Scottish screening programme based on UK NNSC’s advice.	Declined participation	Internal	N/A	N/A	N/A	N/A	N/A
	15. Ethics Review Committee	Review research proposals and/or provide comments and suggestions during hospital board meetings on testing or implementing new digital technologies to improve health and services	Declined participation	Internal	N/A	N/A	N/A	N/A	N/A
**Public and Patient’s representatives**	16. A member of the patient advocacy group or cancer charity	Usually experienced the process of screening and diagnosis	1	External	Low potential	1	MS	Yes	Yes
**Women**	17. Women of breast screening age (e.g., aged 50 year or over)	Invited to attend Scottish Breast Screening Programme for three-yearly mammography	14	External	Low potential	Varied (2 or 3)	Varied (S, MS)	Yes	Yes

Note: N/A = not applicable. *Position: S = supporter, MS = moderate supporter, N = neutral, MO = moderate opponent, O = opponent. See Additional file 1 for description of the analysis, rating and interpretations

Focus group meetings (women, *n* = 14) lasted two hours; participants were aged 59–74 years (mean 65 years). The interviews (*n* = 18) lasted between 26 and 89 min (mean 57 min). Stakeholders’ perspectives were hypothetical and discussed below.

### Influence of stakeholders

Six stakeholder sub-groups had high potential to influence the reform including clinical directors of the Scottish Breast Screening Programme, members of the Scottish Breast Screening Programme Board, hospital management group (e.g., workforce, strategic planning), National Services Scotland, National Screening Oversight Function Board and AI developing company. There were five stakeholder sub-groups with medium potential to influence including readers, members of the National Services Division, hospital management group (e.g., performance, procurement), the Health Technology Assessment group and the Scottish AI Alliance. The remaining four stakeholder sub-groups had low potential to influence including radiographers, innovation research group, women, and patient and public representatives.

Clinical directors had the highest influence on other stakeholders within, and outside the breast screening programme. The Readers (e.g., radiologists) described themselves as ‘AI users’ where AI is implemented rather than as someone with the power to influence the reform.

*‘… the clinical directors of all the centres in Scotland have got the same power, and I think if wanted to bring it (AI) in then we would bring it in,…’* (ID 1).

*‘… we could talk to the government at meetings, but most of us don’t have that power to do so.’* (ID 6).

The members of the hospital management group (high to moderate influence) had several roles including maintaining security, connecting digital equipment and national networks of the entire system; and contributing towards a decision-making process on whether to support the reform or not. As a result, they could influence others (e.g., external stakeholders) and a business case supporting the reform.

*‘If they make it a bigger issue and make it organisational wide my influence would be much, much bigger.… I can absolutely be the executive sponsor for it and such like and absolutely get people rallying behind it.’* (ID 3).

*‘I think I might have some influence in that or be able to bring that (evidence) to the right people’s attention.’* (ID 12).

The National Services Division’s influence involved recommending the reform through their governance channels (e.g., Scottish Breast Screening Programme board, National Screening Oversight Function Board, National Screening Committee and UK National Screening Committee) to the Scottish Government for approval and funding. The National Services Scotland’s influence involved evidence synthesis and communication with other stakeholders based on the evidence. The AI-developing company during this study was already engaged in evaluating an AI system in collaboration with the NHSBSP and had various communications with a range of stakeholders to influence the reform process.

*‘… if the evidence comes through and that’s what we’re looking towards and can fund and can get the business case to support that then yes, we’ll be looking to push it forward’.* (ID 4)

The Health Technology review group had influence in terms of generating evidence (e.g., evaluation of AI in a prospective study) via independent reviews and dissemination of findings for the decision-making process. The influence of the Scottish AI alliance was mentioned as communication of evidence (e.g., AI’s performance in Breast Screening Programme) to other stakeholders via networking and distribution of funds towards generating and testing AI systems. Women and a patient and public representative believed that they could influence other women to attend screening appointments once AI is integrated. However, they thought they had a low influence on the decision of the government on the reform, suggesting the underlying feeling of powerlessness. They were happy to utilise public money or taxes to support the reform. Radiographers and the research project manager also perceived their influence to be very low to impact the reform.

*‘ I think our ability to influence is mixed.… aim is to improve the consistency of how these (technology)things are considered across the service’* (ID 18).

*‘We’re the customer so to go back on your point, we should have a voice.’* (Focus group 1).

*‘I suppose as an ordinary person, you obviously feel quite small in it, and I don’t think you’re going to have much influence. But if you’re particularly interested or passionate about it, you could probably find your way into somewhere to have your influence passed on your behalf’.* (ID 19, patient and public rep)

### Knowledge

Stakeholders’ knowledge varied between ‘none’ to ‘a lot’. Most participants with high-medium influence had a lot of knowledge of AI in health (in general) or the proposed reform and had gained it mainly via conference attendance and work-related meetings where AI in health and/or breast screening were discussed.

*‘What I understand is that through technology and through programming algorithms… it can make an accurate diagnosis based on pre-determined criteria… and potentially more accurately than it can be done by a single individual’.* (ID 7)

Knowledge of stakeholders who had low potential to influence varied widely (e.g., a lot to none). Participants in the women’s group had heard of AI but some of this knowledge was not directly related to health or medicine. Their knowledge was mainly based on reading documents and/or online information after receiving our study invitation. We noted that the members of the patient and public representative struggled to understand the underlying concepts (i.e., utilisation of AI in the screening programme) behind the reform.

*‘I think that the AI term is slightly concerning or could be’.* (Focus group 1)

*‘… to be honest I don’t really know how the computer would look at an image and determine if something look suspicious or malignant* (ID 19, public and patients’ rep).

### Position of stakeholders

Most stakeholders were supporters or moderate supporters of AI. They preferred scenario one (i.e., AI would substitute one of the readers, see Additional file 5) because they either felt it would be easy to implement or they would feel more comfortable if humans were involved in screening.

*I think that’s* (Scenario 1) *quite useful because if the radiologist agreed, then you wouldn’t need a second radiologist, and if the radiologist disagreed, it could go to arbitration, so it’s very similar to the system that we’ve got at the moment, and it would be interesting to see what the AI and you see exactly. I thought would be easiest to implement.* (ID 6).

*I still think you need to have that adjudicator, that human element — any conflict. Even if it’s just completely AI, there still needs to be that human element of checking it and saying, “Wait a minute, let’s double-check it.”* (ID 20).

Moderate supporters wanted to see evidence of AI’s performance (e.g., as good as or better than human radiologists) in the Scottish National Breast Screening Service before they would support the reform strongly.

*‘From a patient perspective, I would almost feel more concerned because there is the potential that a computer could stop my scan ever getting near a radiologist (referring to scenario 2), albeit the prioritisation, they’re kind of wholly reliant on AI. If AI is one of the two sign-offs, so scenario one, I would feel more comfortable with that.… I strongly support it if there’s a robust evidence base, i.e. it’s been proven to operate at least or higher than a consultant… thenaye, yeah, I really support it’.* (ID 16)

*I am getting towards retirement, so I would like to think I keep up with things, and I’m not opposed to change at all. I do think it’s the way forward, but I think we have to do it cautiously until we know where we’re at. (ID 1)*

*In five years’ time ask me again and I’ll maybe have a very different opinion. But I think it’s just… to me it’s not as proven as I would like to say, “Actually yeah, this is brilliant”, but I think it’s so worth exploring… (ID 12)*.

One stakeholder (a radiographer, low potential to influence) self-rated as moderate opponent based on the evidence of AI’s performance in banking, marketing and in the criminal justice system. However, this particular person talked about changing their mind and supporting the proposed reform in the future based on the evidence from prospective studies.

*‘I’m not a fan of AI because… I think when people hear the word, “Intelligence” they assume a level of intelligence but it’s not intelligent in any way… If for example, in the financial world a young couple go for a mortgage but the algorithm, the artificial intelligence and it hasn’t ticked all the boxes then they get, “No”, but actually a human bank manager sitting down and discussing with them knowing them would say, “Yes, we can give you this because we know that…”* (ID 13).

### Alliances

Most stakeholders reported willingness to work collaboratively with other stakeholders (e.g., patients, private companies, and NHS professionals) targeting successful integration of AI into practice. They mentioned about contributing and collaborating during the planning stage of the reform; evidence synthesis in a real setting and/or re-evaluation to judge its impact on the service.

*‘… we work with a lot of industry, we work with lots of different health boards and universities, academia, there’s public sector and Scottish government, we’ll work with anybody. If we can help and make a difference then we’re happy to help’.* (ID 2)

Most of our focus group participants expressed some concerns around working with commercial companies such as AI developers. They believed that to avoid any confusions and bias in findings future prospective studies should be conducted and reviewed by independent researchers.

*‘… when commercial side gets involved and competition and things like that there’s a tendency for shortcuts and maybe even yeah, cutting corners for costs and stuff like that.… I’m talking about the developers, the developers themselves, those who have the control…’* (Focus group 1).

### Interests of stakeholders

All stakeholders were interested in the proposed reform. Advantages and disadvantages of the reform were discussed in relation to its impact on health and the NHSBSP or the entire NHS. Advantages discussed included service efficiency (a) by filling in readers vacancies and reducing work pressure as AI tools would read a large number of mammograms in a short period time; (b) reducing waiting time for receiving mammography results and further assessments (e.g., biopsy) which would further reduce patients’ anxiety and stress; and (c) reducing recall rates (including technical/*’unnecessary’* recalls) which would reduce pressure and stress on the system and patients too.

‘*AI can speed up the screening programme, reduce the load,… so it’s kind of dealing with waiting times et cetera, and it can provide a second opinion when a second opinion might not be available’.* (ID 2)

*‘I think, cutting down recall numbers certainly would save a lot of worry and anxiety amongst the women’.* (Focus group 1)

Other advantages mentioned were that AI would reduce resource use and save money in the future (a) by replacing at least one radiologist with AI would cost less in the long run compared to a human reader; (b) by early diagnosis of cancer and saving lives; and (c) by better managing the overall radiology workflow such as allowing readers to do clinical tasks e.g., seeing patients face-to-face and cancer diagnosis.

*‘… Again costs might come into it I suppose, but I don’t expect it’s going to be as expensive as employing people.* (ID 1)

*‘I think it’s using the manpower better and perhaps with less stress on the system’.* (ID 19)

The main disadvantages mentioned were: (a) AI might be seen as a threat to people’s professional identities and skill sets as humans find change challenging and hence, they might disagree and over-ride AI’s assessment; (b) it might indirectly lead to deskilling human readers by limiting their opportunities to practice reading normal images (as they may focus on abnormal images suggested by AI).

*‘I think it’s probably the fear of the unknown and concern about new things and change and taking a person out of the setup even though there are still humans within the setup.’* (ID 19, public and patients’ rep).

*‘I think there’s a danger well if the radiologist is led by the AI that they become de-skilled actually’.* (ID 12)

Some legal/ethical issues around the reform mentioned were: (a) Public or patients might not accept AI’s errors and then who would take the blame or how to solve these issues; (b) AI might over-diagnose (e.g., recognise lesions at a much earlier stage or lesions of insignificance) or misdiagnose (e.g., misinterprets scars as cancers); (c) there might be some inequalities in detection if AI is not trained appropriately using a wide range of population data (e.g., different ethnic communities, population of different age) and (d) AI might self-regulate by changing the algorithm as it is designed to learn overtime.

*‘… a radiologist can miss something if the machine misses something it would have a very different public perception’.* (ID 3)

*‘If the algorithms are setting the criteria, who takes responsibility and can explain it if there’s a problem?’* (Focus group 2).

Others mentioned that AI could not perform work without human support such as (a) putting parts of a mammogram together might not be possible by AI for a complete assessment or it would not recognise technical issues and hence, might miss some cancers or recall patients unnecessarily; (b) AI don’t have emotions and hence, could not communicate with patients/readers to justify any assessments; and (c) projections of health might not be possible as it could not review the entire health or previous mammograms along with the recent ones. To address these issues all participants suggested keeping humans in the loop at least during the early stages of AI’s integration. Finally, it was believed that AI would read and provide results quicker than the current service, but it might cause pressure on other departments (e.g., surgery) of the NHS for patients’ follow-on treatments.

*‘I think it’s just making sure that we’ve got the whole process there and the timing of the process. We need to make sure we can assess the ladies and obviously then those that are needing treatment can go forward and get the treatment’.* (ID 4)

Most stakeholders believed that AI would bring greater benefits than disadvantages if trained and regulated properly. However, one (moderate opponent) participant compared AI’s integration with ‘Pandora’s box’ and stated that it might create more complications to the service than is anticipated.

*‘In any form of radiology AI is very good at reducing variation because you can ask three radiologists for their perspective on one scan and all three of them can give you entirely different answers, whereas an AI won’t do that’.* (ID 2)

*‘I think it’s Pandora’s box to be honest and I think once it’s opened it’s opened and I don’t think we understand or I don’t think we’re aware of all the ramifications that could come from the use of AI as we bring it in’.* (ID 13)

### The important stakeholders

Thirteen stakeholders were identified as ‘important’ (Table 2 and see methods section for the definition). Three additional stakeholders were included in the list and their views were not explored in the study either because they declined participation or were not approached (e.g., Scottish Government) to take part in this study. Study findings suggested that funding is required from the Scottish Government for the success of the targeted reform and therefore, this stakeholder was added to the list. The UK NSC (see Table 1 for their role) was also added because their approval is needed first for any service reforms involving screening programme in the UK. The NSC and the Scottish Government also rely on their advice in deciding funding and national approval. The NSC should be added because they have influence on other stakeholders on a national (i.e., Scotland) level. Women (low potential to influence) were considered as ‘important’ because these stakeholders are the main users of the breast screening service. Moreover, most stakeholders mentioned that any reform proposals must be communicated to, and approved by, the women as they might, otherwise, not turn up for their mammography.

**Table 2:** List of agreed important stakeholders

*Scottish Government
*UK National Screening council/committee (UKNSC)
*National (Scottish) Screening Council (NSC)
National Services Scotland (NSS)
National Screening oversight Function Board (NSO)
National Services Division (NSD)
Scottish Breast Screening Programme Board
Clinical directors of Scottish BSP
Readers (radiologists or qualified radiographers who can assess mammograms)
Hospital management group
Public and patients’ representatives
Women
AI developing company

* While they were identified as important stakeholders based on the information gathered, they either declined or were not approached to take part in this study

### Strategies to improve future support

Most of our stakeholders were in support of the proposed reform. Hence, five strategies were generated to maintain their support and increase their power and leadership targeting the reform. Below is a summary of the proposed strategies with a full description available in Additional file 6.

### Strategy 1: improve knowledge of AI for all stakeholders

Most stakeholders (including internal and those with low to high potential to influence) did not fully understand the process and impacts of the proposed reform or sometimes found it controversial. They believed further information (e.g., based on evidence from prospective studies) would improve their confidence and support of the reform. Some requested a lot of information (e.g., facts and figures about AI’s performance, and consequences). Others (e.g., women) requested ‘little’ and ‘balanced’ information as they believed that their capacity to understand the entire reform process was limited, or they had the trust that the best service would be recommended by the NHS. Lastly, this information should be available to all stakeholders including patients, to assist them in making an informed choice when AI is integrated into practice.

### Strategy 2: improve skills and ability of stakeholders directly involved in delivering the service

Stakeholders (e.g., readers, NSD; high and medium potential to influence) who provided direct services as part of the Scottish Breast Screening Programme also requested further information related to their roles to increase their confidence, ability and support of the reform: what AI can or can not do, how AI works and its impact on the system; how to interpret AI’s assessment of mammograms and what responsibilities they or readers must have for ‘safeguarding the system’ to make sure the service is running as intended and safe for all.

Some suggested that future staff training would be required to improve their ability to successfully embrace AI into practice. However, they were unsure about the types of training and believed that during a prospective study any gaps in their skills and training needs could be identified. However, user-friendly training modules (e.g., video or demonstration by a colleague) were suggested for an easy transition.

### Strategy 3: empower women to influence the screening service

The majority of the Scottish Breast Screening Programme’s service users are women who had low potential to influence the proposed reform. Women and the public and patient representative thought that they had a voice and power to influence other women to attend (or not) their screening appointments. Therefore, it was believed that empowering these stakeholders further by turning their knowledge into action (e.g., using the media, posters) or experience into voice (e.g., showcasing personal testimonies through cancer charities’ websites) might be useful for influencing other service users or decision-makers. Generating and promoting such communication using case studies, television interviews, and podcasts were suggested. Social media was proposed as a major platform for these activities. However, a few stakeholders had some reservations about the use of social media because they believed that negative comments or blogs could damage the public perception of the reform and reduce support.

### Strategy 4: use a collaborative team-based approach involving stakeholders to address challenges and improve support

Various context and deployment-related challenges were mentioned by out study participants. One of the main challenges suggested was having a wide range of stakeholders involved in the reform process and that lack of communication across health boards and different partners. As a result, reform proposals often take long to get approved or sometimes approval is not given because all stakeholders were not involved at the planning stage or were unaware of the entire process. Other challenges mentioned were around the AI-related IT and emergency services: no nationwide IT infrastructure is available to integrate and connect different software or digital equipment and mobile units, and who/how (e.g., ‘*an AI guru’*) should be contacted to address technical problems. Therefore, a collaborative and team approach were proposed to engage relevant stakeholders (i.e., who would involve deploying and sustaining related tasks) from the beginning to the entire cycle of the reform process to improve its acceptance and continuous support. It was further suggested to use a visual model to explain and discuss the reform process with relevant stakeholders so that they could think through the process and identify potential issues and solutions. A need for a ‘champion’ was also highlighted to lead the teamwork targeting a successful reform.

### Strategy 5: improve real-world evaluations of AI

All stakeholders suggested seeing evidence of AI’s performance from a prospective study (e.g., a ‘blinded randomised’ trial with long-term follow-up) and comparing it to the current service of the Scottish National Breast Screening Service and/or NHSBSP would be beneficial. Stakeholders’ views were captured further regarding what sort of evidence would satisfy them to continue and increase their support in the future (Additional file 6).

## Discussion

This, to our knowledge, is the first published study that conducted a stakeholder analysis targeting a real-world evaluation of integrating AI algorithms into a breast screening service. Worldwide health services are facing workforce issues to provide patient care like the NHSBSP in the UK. While the specific stakeholders may not always be directly comparable across regions or countries, limiting the generalisability of this work, the types of issues identified are likely to be broadly similar across breast screening programmes, especially concerning attitudinal aspects.

We captured a range of views from a wide variety of stakeholders. Most stakeholders were in favour of the reform (i.e., integrating AI algorithms into the Scottish National Breast Screening Service for breast cancer detection) but their knowledge and interests varied. They were keen to collaborate with other stakeholders for AI’s successful integration into the screening service. They expressed some concerns towards using AI and hence, suggested keeping human readers involved until they are confident that AI’s performance is satisfactory in the real-world setting of the NHSBSP. Stakeholders’ importance and influence were also assessed to guide future activities in this area.

The stakeholders (e.g., professionals) who were approached and/or took part in this study were mostly the leads of relevant organisations. Some frontline implementers (from the professional group) were unable to participate despite repeated efforts to reach them and of whom, some provided reasons for not taking part such as lack of time due to other commitments.

Our study captured stakeholders’ views of what evidence should be generated and what should be considered when evaluating AI in prospective studies. At the time of this study, no published prospective study was reported with long-term follow-up nor any evaluation framework for AI systems was available. The National Institute for Health and Care Excellence (NICE) subsequently published a medtech innovation briefing (MIB) on AI in mammography to highlight the importance of assessing impact of such reform on potential patients and the system [4]. It also emphasised the importance of generating guidance for the evaluation of components and the processes to be used during such assessments. In addition to this briefing, eight objectives towards evaluation were suggested by the AI in Health and Care Award playbook [23]: including establishing the accuracy, safety, effectiveness, value, fit with site, feasibility and suitability of scale-up, and implementation considerations. On comparison, the findings of our study are in line with these proposed objectives.

Most studies in the literature conducted surveys and/or interviews to explore views of the patient and public on the use of AI in mammography or medical care or radiology in general [24–27]. Findings from our focus group study are comparable with these studies highlighting that the screening population approves the introduction of AI systems for disease detection as long as there is some human involvement throughout the reading (e.g., 1st or 2nd reader) and/or diagnosis process.

Stakeholder analysis has been conducted worldwide [28–33] as part of health reform activities, but the methods and steps used are found to be heterogeneous [17, 18]. Most studies discussed the implications of the analysis they conducted and related findings, and only a few publications explained future actions such as strategies to engage with stakeholders [34–38] or recommendations to improve their support [39, 40]. Our study was drawn on WHO provided instructions and tools supported by academic theory and real-world application [20]. This practical tool helped us to identify and assess the stakeholders and their complex characteristics in a rigorous, transparent, and systematic manner. This study article contributes to the literature of stakeholder analysis and scholarly research by advancing the knowledge of the theory and analysis processes by providing the first detailed description of the methodology for every step of the WHO guidelines. This article therefore presents practical guidance on how we adopted the tools provided by the guide, how we scored and/or interpreted the findings with examples and finally, how we used the overall findings to inform strategies for managing ‘important’ stakeholders.

Some of the strengths of this study were that we used a systematic approach from the pre-selection of relevant stakeholders to data collection, analysis, interpretation and development of strategies to manage ‘important’ stakeholders. The research team managed to successfully engage with the majority of the targeted stakeholders during the post-covid time period and generated rich data. Several measures were taken to enhance the trustworthiness of the data. For example, the topic guides, scenarios and checklist and analysis tools were pilot tested before data collection. Interviews and focus group meetings were transcribed verbatim by an authorised and expert external transcriber company and were double checked by a researcher before the coding process began. Two researchers double coded 10% of the transcripts to inform and finalise the coding guide for the purpose of consistency and replication of the process. An analysis guide was developed, and we explicitly stated the criteria used for assessing characteristics to minimise bias and reduce ambiguity.

Some limitations include limiting the data collection to three geographical areas of Scotland. However, these areas cover large mixture of urban and rural population in relation to the breast cancer screening programme in Scotland. Due to the nature of the study (i.e., hypothetical and qualitative) it might be that some views were not captured or only people who are in support of the reform agreed to take part in the study. Efforts were made to identify and recruit relevant stakeholders during the entire cycle of the study. For example, we used snowballing technique during the data collection process to identify stakeholders.

who were not in our list and who might support or oppose the reform. As a result of this, one moderate opponent was recruited in this study. Perspectives of some stakeholders such as government officials were not captured. As per example, members of Scottish Government were not invited to take part in this study because of the hypothetical nature of this study and researcher’s limited access to them during the COVID pandemic. Women aged 59–74 year took part in the focus groups and hence, views of younger women (e.g., less than 50 year old) were not captured. Future studies should consider capturing younger women’s views and empower them to influence the future reform. The analysis was conducted by one researcher only, but the analysis tables and findings were reviewed by the working group and agreed by the workshop participants.

## Conclusion

In our study, stakeholders were multiple, and they showed interest in various activities targeting the reform (i.e., integrating AI algorithm into the Scottish National Breast Screening Service for breast cancer detection). Besides some concerns were raised, they were mostly supportive of using AI in the breast screening programme. Five strategies were developed to maintain and improve the support of ‘important’ stakeholders. Findings of this study might contribute to shape the AI integrated future breast screening service.

In the next stage of the study, the proposed strategies must be co-produced with relevant stakeholder groups. Challenges related to implementing the strategies may include allocating resources such as time, funding, and staff, the organisational structure and individuals involved, and collaboration within the organisations (e.g., mutual communication and multidisciplinary codesign). Therefore, perceived challenges must be identified through discussion with the working group and relevant stakeholder groups and tackled throughout the implementation process to ensure its compelling design and effective delivery targeting the proposed reform. Future research also needs to evaluate the effectiveness and impact of the strategies once implemented, which would further contribute to advance the field of health reform policy implementation work. This article provides guidance for a novel approach to aid future researchers, policymakers or health planners to conduct similar studies targeting healthcare reform.

### Electronic supplementary material

Below is the link to the electronic supplementary material.

Supplementary Material 1

Supplementary Material 2

Supplementary Material 3

Supplementary Material 4

Supplementary Material 5

Supplementary Material 6

## Data Availability

All data generated or analysed during this study are included in this published article and its supplementary information files.

## Abbreviations

AI: Artificial Intelligence
iCAIRD: The Industrial Centre for Artificial Intelligence Research in Digital Diagnostics
NHS: National Health Services
NHSBSP: National Breast Screening Programme
NSC: National (Scottish) Screening Council
NSS: National Services Scotland
NSO: National Screening oversight Function Board
NSD: National Services Division
NICE: National Institute for Health and Care
NICE: Excellence
UK: United Kingdom
UKNSC: UK National Screening council/committee
WHO: World Health Organisation
