# Supplementary material for: A stakeholder analysis to prepare for real-world evaluation of integrating artificial intelligent algorithms into breast screening (PREP-AIR study): a qualitative study using the WHO guide
Source: BMC Health Serv Res. 2024 May 2;24:569. doi: 10.1186/s12913-024-10926-z (PMC11067265; doi:10.1186/s12913-024-10926-z)
Supplement: Supplementary file 1 — Supplementary Material 1 [file 12913_2024_10926_MOESM1_ESM.docx]

| **Study Objectives** | **Steps involved** |
| --- | --- |
| 1. Identify possible stakeholders | 1. Define the purpose of the study and develop a plan (i.e., protocol, working group) 2. Compile and review existing information on stakeholders 3. Finalise the list of possible stakeholders and prioritise stakeholders with input from experts. |
| 1. Explore stakeholders’ perspectives and describe their characteristics | 1. Adapt the tools 2. Gather information  - Recruitment - Data collection and management - Focus group meeting with women - Interviews with ‘professionals’ and Patient representatives  1. Analysis |
| 1. Prioritise stakeholders in terms of importance | Discussion of findings (from objective B) with the iCAIRD team (involving health data scientists, health services and NHS researchers, radiologists, physicists, and project managers) to finalise the ‘important’ stakeholders’ list. |
| 1. Develop strategies to manage ‘important’ stakeholders | Development of strategies and discussion with the iCAIRD team (involving health data scientists, health services and NHS researchers, radiologists, physicists, and project managers) to finalise the strategies. |

**Additional file 1.** **Outline of the process used for stakeholder analysis and informing strategies.**
