# Supplementary material for: A stakeholder analysis to prepare for real-world evaluation of integrating artificial intelligent algorithms into breast screening (PREP-AIR study): a qualitative study using the WHO guide
Source: BMC Health Serv Res. 2024 May 2;24:569. doi: 10.1186/s12913-024-10926-z (PMC11067265; doi:10.1186/s12913-024-10926-z)
Supplement: Supplementary file 2 — Supplementary Material 2 [file 12913_2024_10926_MOESM2_ESM.docx]

A combined table for stakeholder characteristics (including their description), instructions for scoring and filling in the stakeholder table and reference chart (question numbers that pertain to each column on the stakeholder table)

| **Stakeholder characteristics (as in the WHO guide)** | **Description and analysis/rating** | **Associated topic guide questions (question reference number)** | |
| --- | --- | --- | --- |
|  |  | **For ‘Supporters’, if relevant** | **For ‘Opponents’, if relevant** |
| I.D. number | A unique identification number (I.D.) is given to each stakeholder. | | |
| Position & organisation | Their job title, role and the organisation they work for | | |
| Internal/external | Internal stakeholders work within the NHS organisation that is promoting or implementing AI systems; all other stakeholders are considered external. | | |
| 1. Knowledge | The level of accurate knowledge the stakeholder has regarding the proposed reform under study and how each stakeholder defines it.  This characteristic is divided into two parts. The first part is the level of accurate knowledge the stakeholder has regarding the reform.  **Analysis:** Knowledge should be rated from 3 to 1 (and reviewed to ensure consistent scoring): 1= none, 2= some and 3= a lot of knowledge. | #1, #2 | |
|  | The second part is to record how each stakeholder defines the proposed reform and captures their own words. | #3 | |
| 1. Position | The position is the key to establishing whether or not the stakeholder will impact the reform. Position refers to the stakeholder's status as a supporter, neutral or opponent of the reform: S (supporter), MS (moderate supporter), N (neutral), MO (moderate opponent), O (opponent).  The position of the stakeholders as perceived by others is also to be entered here with reference to the ID number of the person who stated that opinion.  **Analysis:** Stakeholders who agree with the reform are considered as supporters (S), those who disagree are considered opponents (O), and those who do not have a clear opinion or whose opinion could not be discerned are considered neutral (N) and similarly who express some but not total agreement or opposition are considered as moderate supporters (MO) or opponent (MO).  A combination of these two pieces of information (self-reported and other stakeholders' opinions) informed the final position of a stakeholder, and it is rated as S, MS, N, MO, and O. | #7, #8 #9, #10, 14  and  #15, #16, #17 | #7, #11, #12, #13, #14  and  #18, #19 |
| 1. Alliances | It’s about a union or relationship; alliances are formed when two or more organisations collaborate to meet the same objective e.g., support or oppose the reform.  **Analysis:** Any organisations mentioned by stakeholders as they usually work with and/or would prefer to collaborate with to support or oppose the reform should be entered here. | #9a, #9e, #9c, #9g | #12c, #12 a, #12e, #12g |
| 1. Interests | The stakeholder’s interest in the reform or the perceived impact (advantages/disadvantages) that the reform may bring to the stakeholder or his or her organisation. Findings from this characteristic help to better understand his or her position and address any concerns.  **Analysis:** Information provided by stakeholders should be entered in as much detail as possible, and a general conclusion should be drawn based on the expression of the stakeholder. | #4, #5, #6 #8, #10 #14 | #4, #5, #11, #13, #14 |
| 1. Resources | It is about a source of support or aid for a stakeholder. Resources are of many types such as political, financial, and various expertise such as technical and technological. It is an important characteristic that is summarised by the power index and it helps to determine the level of force with which the stakeholder might support or oppose the reform.  **Analysis:** It has two parts: the number of resources (quantity, **A**) a stakeholder has within his or her organisation or area, and the ability to utilise those resources (**B**) to influence the reform. The details based on our study are discussed below-   1. **Quantity (A): the details of different resources discussed by our stakeholders are discussed below -** 2. **Human/skills** (manpower with skills required for the mammography and reading images) 3. **Technical=** It is the capacity  - to produce guidance for good practice in the area of AI development/deployment, - to produce training and related materials for building up skills and capabilities to use AI in health and - contributing towards the planning stage in relation to AI’s implementation by evidence synthesis, such as being involved in clinical trials or systematic assessments (e.g., via health technology assessment process) and/or contributing to the business case by providing comments/suggestions, modelling a workforce plan  1. **Technologica**l= It is the capacity to accommodate, integrate or maintain the system/process as part of the reform e.g., IT, data infrastructure, quality control, addressing any issues in the area of technology, including AI 2. **Political (i.e., influential) =** It’s the ability to lead, gather support from others or influence others for/against the reform via action or communication e.g., by direct chat, attending meetings/conferences and disseminating information/evidence of AI in health/breast screening programme. 3. **Financial** **=** It is about if a stakeholder has possession (ownership) and/or control of financial resources or one has the ability to seek funds through their network and/or by producing/supporting a business case.   **Scoring of quantity** (review to ensure consistent scoring):   - 3= more than two types of resources - 2= two types of resources - 1= less than two types of resources and - 0= none  1. **Ability to mobilise/utilise resources (B) = It reflects whether stakeholders could decide (or not) as an individual or as one of several persons from their organisation (e.g., NHS) to utilise the resources to make the reform happen in reality.**   **Scoring for ability**:   - 3= stakeholders can make the decision for resource utilisation. - 2= stakeholder is one of the several people that can make decisions on resource utilisation. - 1= stakeholders cannot make decisions regarding the use of the resources. | #9a, #9c, #9e, #9f  #9a, #9b, #9c, #9d, #9e | #12a, #12c, #12e, #12f  #12a, #12b, #12c, 12d, #12 e |
| 1. Power | Power is defined as the combined measure of the number of resources a stakeholder has **(A)** and his/her capacity to utilise them **(B).** It is the ability of a stakeholder to affect the reform. In addition to the guidance of WHO, we collected stakeholders’ self-statement about their influence/power to affect the reform. We combined both findings to interpret their influence: whether a stakeholder has low/moderate/high potential to affect the reform under study.  **Analysis**  **Power= Average score of resources (from above/section 5)**  **= (A + B) / 2**    **Scoring for power**   - Total score 1 (less than 2) = stakeholder/organisation has low power - Total score 2= stakeholder/organisation has medium power - Total score 3 (more than two) = stakeholder/organisation has high power - 0 = stakeholder/organisation has no power to affect the reform.   Example of Power calculation and interpretation  **Power = (3 +2)/2**  **= 2.5** (when the score is more than two, it means having high power)  Where, A (3) = more than 2 types of resources (e.g., technical, technological and financial) and  B (2) = stakeholder is one of the several people that can make decisions on resource utilisation  Final interpretation: If a stakeholder mentioned (self-statement) that he/she has ’a lot’ of influence on the proposed reform, then the power was interpreted as having high power based on both findings. | #14 (self-statement) + Resources (as above/section 5) | |
| 1. Leadership | It is defined as the willingness of the stakeholder to initiate, convoke or lead an action to shape opinion for or against the reform. It is because of their ability to build partnerships and motivate others and/or their charismatic authority, personal commitment, and motivation.  **Analysis: Interpretation**   - Yes = stakeholder has leadership - No = stakeholder is not willing to initiate and/or lacks it. | #9a, #9b, #9c, #9e | #12a, #12b, #12c, #12e |
| 1. Power and leadership analysis | It is the key aspect of a stakeholder analysis to indicate their level of influence. The grouping below is made by combining interpretations from Power and Leadership analysis (as above).  Interpretation of findings   - Group 1 = high potential to influence, who have leadership and high power - Group 2 = moderate potential to influence, who have leadership and medium power and - Group 3 = low potential, who have no leadership but high to medium power. | Not applicable | Not applicable |
