# Supplementary material for: A stakeholder analysis to prepare for real-world evaluation of integrating artificial intelligent algorithms into breast screening (PREP-AIR study): a qualitative study using the WHO guide
Source: BMC Health Serv Res. 2024 May 2;24:569. doi: 10.1186/s12913-024-10926-z (PMC11067265; doi:10.1186/s12913-024-10926-z)
Supplement: Supplementary file 3 — Supplementary Material 3 [file 12913_2024_10926_MOESM3_ESM.docx]

Date:

Location:

ID

Opening Question: Firstly, what is your job title? How long have you been working as a …?

| Leading questions | Possible prompts |
| --- | --- |
| 1. Have you heard of using ‘artificial intelligence’ (AI) for disease detection (e.g. reading X-ray images)? |  |
| 1. If so, how did you hear of it? |  |
| 1. What is your understanding of ‘Artificial Intelligence’ enabled/led breast cancer detection? | How an AI system could help detect breast cancer? |
| 1. What do you think are the benefits of using AI systems for breast screening? | Any benefits to your organisation/patients? |
| 1. What are the disadvantages of using AI systems for breast screening?  - Do you have any comments about the impact of using AI in terms of job security for NHS Staff)? | Any disadvantages to your organisation/patients?  What solutions would you suggest solving the issues? |
| 1. What outcomes would be important while using AI in breast screening service? Ask specific outcomes for patients (Additional question) |  |
| 1. Which of these categories best describes your opinion on the utilisation of AI systems for breast screening in general? 2. I strongly support it 3. I somewhat support it 4. I do not support nor oppose it 5. I somewhat oppose it 6. I strongly oppose it   *For those who answer a, b or c continue below*. *If answered d or e plz go to Q 11-13* |  |
| 1. Which of the two types of AI systems use do you support? 2. Scenario 1: as an independent second reader 3. Scenario 2: as a soft triage reader 4. Ask if they have any other suggestions (scenarios) regarding how AI could be used in breast screening programme (BSP). |  |
| 1. For those aspects (from Q7- highlight the one they support) of AI systems use that you do support: 2. For this scenario … your support, can I ask in what way you would demonstrate this support? 3. Would you take the initiative in supporting this type of AI system use in (your) screening practice, or would you wait for others to do so?  - Who are the others? - What kind of initiatives would you take? What would be the outcomes of these initiatives be? - Do you have any other concerns that may stop you from taking the initiative to support it?  1. What resources would be required to support this initiative (AI use in breast screening)? 2. From your knowledge, what resources are available now, and how quickly can they be mobilised/used for implementation efforts?  - Human/financial/technological/political/other (prompts) - What other supports would you need in relation to its successful implementation into practice?  1. Can you express this support openly in public? (if asked before, don’t repeat) 2. What conditions/evidence (scientific) would have to exist for you to express this support? 3. Would you ally (work) with any other persons or organisations in these actions to support the AI system implementation?  - Which partner do you usually work with and in what way?   OR (What evidence would you need to see regarding AI’s success in real settings?) |  |
| 1. Under what/other conditions would you choose NOT to support this AI system?   Additional questions  Why do you not support the other scenario?   - Do you think one of the scenarios would work better than the other during the pandemic to tackle workload? Which one and why? - Do you think one of the scenarios would work better than the other after the pandemic to improve future care/services? Why/why not? - Have you got any comments on the other scenario (that you don’t support) | Any other concerns?  If yes, could you suggest some possible solutions? |
| 1. Which of the following aspects of these AI systems do you oppose? Why?   a) Scenario 1: as an independent reader  b) Scenario 2: as a soft triage reader |  |
| 1. For those aspects that you oppose: |  |
| 1. In what manner would you demonstrate this opposition? Why? |  |
| 1. Would you take the initiative in opposing this type of AI system, or would you wait for *others* to do so?  - Who are the others? | Have you got any other concerns that would discourage you from supporting an AI system? |
| 1. (If you were a supporter) Do you have *financial* or *human resources* or *time* available to implement this type of AI system? |  |
| d. From your knowledge what resources are available and how quickly can they be mobilised?  - Any other resources/support would be required? | Human/financial/technological/political/other |
| e. Would this opposition be public? |  |
| 1. What conditions/evidence would have to exist for you to express this opposition? | What other information/evidence would you look at to express your opposition openly? |
| 1. Would you ally with any other persons or organisations in these actions to oppose the AI system implementation? | Which persons/organisations? Why? |
| 1. Under what conditions would you come to change your mind and support this AI system? | What other/evidence would be required to satisfy you to support AI?/ |
| 1. How would you best describe the extent of your influence over the application/implementation of AI systems for breast screening Programme? By influence, we mean the power to have an effect on this AI system being used OR to affect or change how it’s used. (Additional question) | |
| Q15-17 for supporters/neutral (i.e., who answered a, b or c for Q7)   1. What other persons or other organisations do you think would support the implementation of AI systems for breast screening? 2. Are you aware of anyone who has already demonstrated support? If yes, how was this support demonstrated? (e.g. openly in meetings/in public/email communication) 3. What do you think these supporters would gain from this implementation (use of AI in the Breast Screening Programme)?  - Which of these supporters do you think would take the initiative to actively support/influence this implementation? | |
| Q18-19 for opponents (i.e., who answered d or e for Q7)   1. What other persons or other organisations or departments within your organisation do you think would oppose the implementation of AI systems for breast screening?  - Are you aware of anyone who has already demonstrated any objections towards AI use? If yes, how was this objection demonstrated? (ask for names/roles)  1. What do you think these opponents would gain from preventing implementation? Any suggestions regarding how to turn them into supporters? | |
| Close-up questions   - What do you think are the key challenges in the health sector that you might come across as a barrier (towards implementing AI)? - What recommendation can you provide to improve the success of AI’s implementation in Breast screening? - What are the facilitators towards digitising BSP successfully using AI? - Any other factors that you think might be important that we have not covered? | |
