# Supplementary material for: A stakeholder analysis to prepare for real-world evaluation of integrating artificial intelligent algorithms into breast screening (PREP-AIR study): a qualitative study using the WHO guide
Source: BMC Health Serv Res. 2024 May 2;24:569. doi: 10.1186/s12913-024-10926-z (PMC11067265; doi:10.1186/s12913-024-10926-z)
Supplement: Supplementary file 4 — Supplementary Material 4 [file 12913_2024_10926_MOESM4_ESM.docx]

Additional file 4: A topic guide for focus groups and interviews of patient and public rep

| Questions/prompts |
| --- |
| 1. Have you heard of using ‘artificial intelligence’ (AI) for disease detection (e.g. by reading X-ray images)? |
| 1. If so, how did you hear of it? |
| 1. What is your understanding of how AI could support breast cancer detection?   Additional questions  - Do you feel more information would be helpful in improving your knowledge?  - If yes, what information would be helpful?  - What information/evidence would give you the confidence to trust AI to read your mammogram and make a diagnosis?  -Any suggestion where this information should be available?  - How would you like to receive this information? (what format- paper copy/online version/via webinar?) |
| 1. What do you think are the benefits of using AI systems for breast screening? Any benefits to you/NHS |
| 1. What are the disadvantages of using AI systems for breast screening? Any downsides to you/NHS/other |
| 1. What outcomes would be important while using AI in breast screening service? Ask specific outcomes for patients (Additional question) |
| 1. Which of these categories best describes your opinion on AI use for breast cancer screening? 2. I am strongly in favour of using it 3. I am somewhat in favour of using it, but I have my reservations about it (e.g. it depends on …) 4. I feel indifferent about its use (I don’t support nor against its use) 5. I am somewhat against it, but it depends 6. I am strongly against it   *For those who answer a, b or c continue below*. *If answered d or e plz go to Q 11-13* |
| 1. Which of these two types of AI system use do you support? and why?   a) Scenario 1: as an independent second reader  b) Scenario 2: as a soft triage reader  c) Ask if they have any other suggestions (scenarios) regarding how AI could be used in the breast screening programme (BSP). |
| 1. Imagine that you are being invited, as a part of a routine breast cancer screening practice that we have in Scotland, to have your mammography done. Now, reading your mammograms may involve the use of AI - in the way you were in favour of (As a second reader/Triage): 2. Would you be happy for the public money to be used for training AI systems for breast screening at NHS Scotland?   e.g. (diverted from training healthcare professionals to training AI systems)#   1. Would you be happy for AI to read (the scenario you support) your mammograms as soon as it’s rolled out or would you rather wait for it to be used for a while and pass the test of time?  - With whom would it have to ‘pass’ that test (other patients or clinicians)? - Do you have any other concerns that would stop you from using it straight away?  1. Do you feel like you have any influence on the decision of the government or local authorities to utilise AI systems for breast screening and/or to mobilise such resources (public money)?  - How would you best describe the extent of your influence? Have you been involved in such activities previously- influencing the implementation of any new rules/policies in health?  1. What other support/resources would be required for AI’s successful implementation? 2. If invited to openly/publicly express how you feel about the use of AI for breast screening, would you be happy to do it? 3. What information/evidence would give you the confidence to trust AI to read your mammogram and make a diagnosis? 4. Would you join with any other persons or organisations in these actions to support/oppose the use of the AI systems?  - Which persons/organisations? Why? - For example, patient advocacy groups, AI system developers, or scientists |
| 1. Under what/other conditions would you come to change your mind and not ACCEPT this AI system being used to read your mammograms? 2. Which of the following aspects of these AI systems do you oppose? Why?   a) Scenario 1: as an independent reader  b) Scenario 2: as a soft triage reader |
| 1. a) In what manner would you demonstrate this opposition? Why? 2. Would your decision be final about not supporting AI’s involvement in breast screening without knowing how other people feel about it?   or  Would you say your decision be final only if you knew that others shared your feelings?   - Who are the others (patients/clinician)?   -Do you have any other concerns related to AI and its application to detect breast cancer?  - If yes, are there any possible solutions to these concerns- can you think of them?   1. Would you rather support utilising extra public resources (tax money) for alternative ways to tackle the current workload situation of the breast screening unit or support the use of suggested AI systems to tackle the current situation?  - By alternative ways, we mean bringing more radiologists who are trained abroad or locum radiologists (who temporarily fulfil duties of others in the area)– these could be much more expensive to support by NHS.  1. What other support/resources would be required for AI’s successful implementation? If you were a supporter, what resources would you have to support this implementation? 2. Would this opposition be public? 3. What evidence would you need to see regarding AI’s success in real settings?  - What other/evidence would be required to satisfy you to support AI?  1. Would you ally with any other persons or organisations in these actions to oppose the AI system implementation?  - Which persons/organisations? Why? |
| 1. Under what/other conditions you would you come to change your mind and ACCEPT this system being used to interpret/read your mammograms? |
| 1. How would you best describe the extent of your influence over the application/implementation of AI systems for the breast screening Programme? By influence, we mean the power to have an effect on this AI system being used OR to affect or change how it’s used. (Additional question) |
| Q15-17 for supporters/neutral (i.e., who answered a, b or c for Q7)   1. What other persons that you know personally or in general, or other organisations do you think would be in favour of AI systems being used for breast cancer screening?   (Prompts: Other women that you know, female sub-populations (working/retired, older/younger, living in cities/rural areas), healthcare professionals (GPs, radiologists), clinical organisations (patients advocacy groups), government?) |
| 1. Are you aware of anyone who has already demonstrated support? |
| 1. What do you think these supporters would gain from the application of this AI system into practice OR lose from not being able to use it?  - Which of these people/groups do you think would take the initiative actively to support the AI system uptake for breast cancer screening and what actions could they take (any thoughts)? |
| Q18-19 for opponents (i.e., who answered d or e for Q7)   1. What other persons you know personally or in general or other organisations, do you think would be against using AI systems for breast cancer screening?   (Prompts: Other women that you know, female sub-populations (working/retired, older/younger, living in cities/rural areas), healthcare professionals (GPs, radiologists), clinical organisations (patients advocacy groups), government?) |
| 1. What do you think these ‘opponents’ would gain from stopping AI application in breast screening practice OR lose from its use? Any suggestions regarding how to turn them into supporters? |
| Close-up questions   - Do you have any other comments/suggestions you would like to make about using AI systems for breast cancer screening? - Any other factors that you think might be important that we have not covered? |
