# Supplementary material for: A stakeholder analysis to prepare for real-world evaluation of integrating artificial intelligent algorithms into breast screening (PREP-AIR study): a qualitative study using the WHO guide
Source: BMC Health Serv Res. 2024 May 2;24:569. doi: 10.1186/s12913-024-10926-z (PMC11067265; doi:10.1186/s12913-024-10926-z)
Supplement: Supplementary file 5 — Supplementary Material 5 [file 12913_2024_10926_MOESM5_ESM.pdf]

A study of people's views on using artificial intelligence (AI) systems for breast cancer screening

- This document includes a description of the NHS Breast Screening Program (BSP) in Scotland, how mammograms (x-ray pictures of the breast) are currently interpreted in it and two fictional scenarios of how AI systems may change it.
- We would like you to reflect on those two scenarios.
- During your group discussion (or interview) we will ask you specific questions about your preferences and opinions on the proposed scenarios.

1 in 8 women in Scotland will be diagnosed with Breast Cancer at some point in their lives.

Breast screening aims to find breast cancers early. It saves 130 lives every year in Scotland.

All women in Scotland aged from 50 to70 who are registered with a GP are automatically invited by letter to a static or a mobile centre for breast cancer screening every 3 years.

Currently, two radiologists check each woman's mammograms for any abnormalities. In rare cases where they disagree, a third radiologist assesses the images to finalise a decision on the diagnosis **(Diagram 1 below)**.

The results are sent to the GP no later than 2 weeks after the appointment. After screening, about 1 in 25 women will be called back for further assessment and 1 in 4 of them are diagnosed with cancer.

8% of hospital posts are currently unfilled and outsourcing costs for radiology have doubled in Scotland between 2017 and 2018.

Diagram 1. Current image interpretation process within the NHS Breast Screening Program

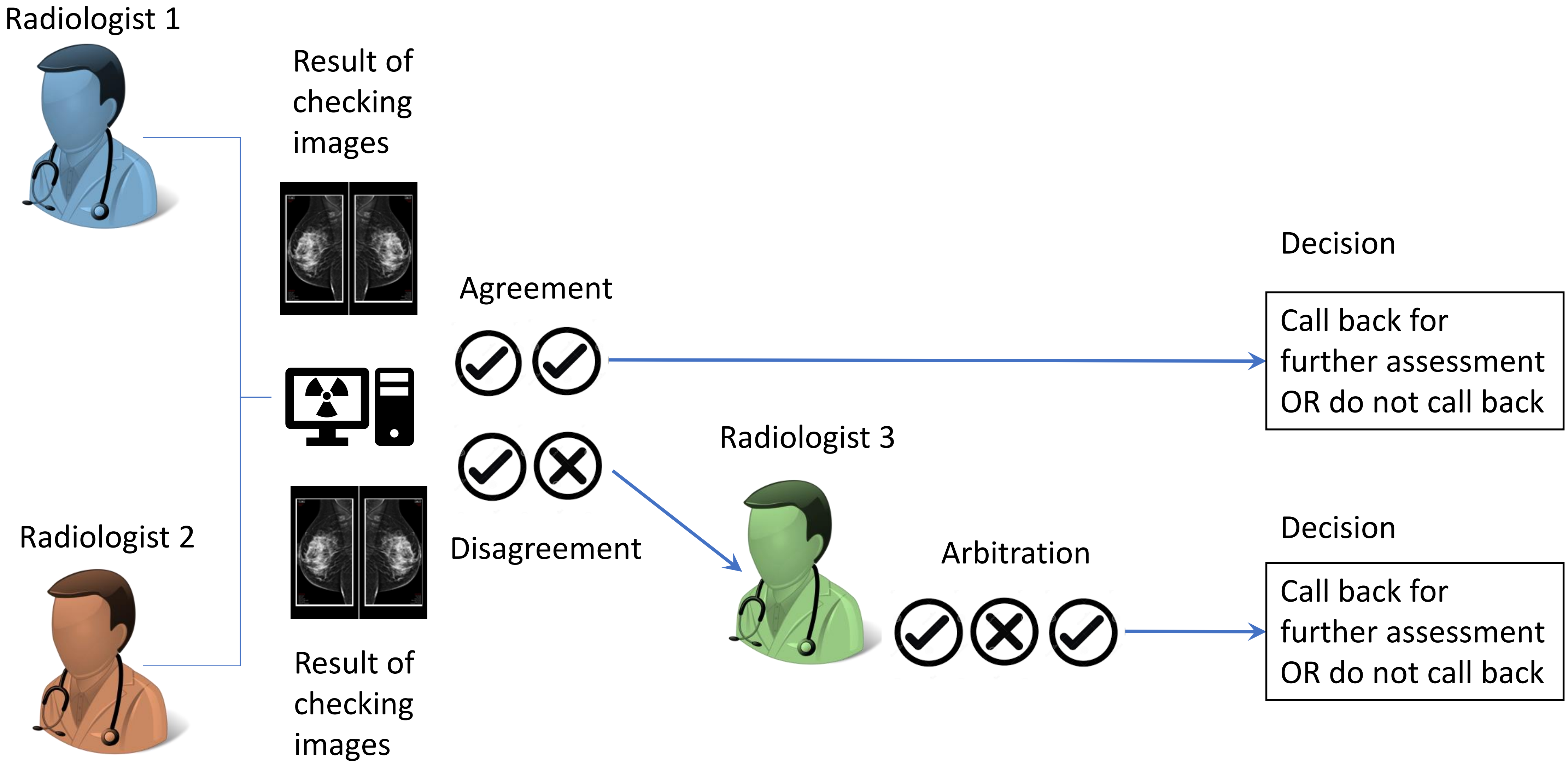

An artificial intelligence (AI) system has been tested on a set of existing mammograms from the Breast Screening Programme (BSP) in the North East of Scotland. The results from this test showed that this technology was as good as two radiologists. It is expected that its use in real world could eventually speed up diagnosis, as it will be capable of detecting cancers earlier than the current workflow. It could also help to reduce numbers of women called back for assessment and invasive tests like biopsies.

We would like you to imagine two fictional scenarios of this AI system becoming a part of the BSP in Scotland:

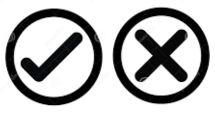

Scenario 1: An AI system as a second interpreter of mammograms, substituting one of the two radiologists who would otherwise do this work. Another radiologist would be available to arbitrate a disagreement between the radiologist and the AI system.

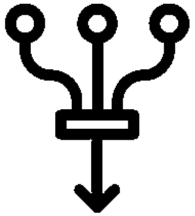

Scenario 2 : An AI system would look at all breast screening images and flag up the ones that a radiologist should look at first (for example most urgent cases).

Scenario 1: AI would substitute one the readers (radiologists)

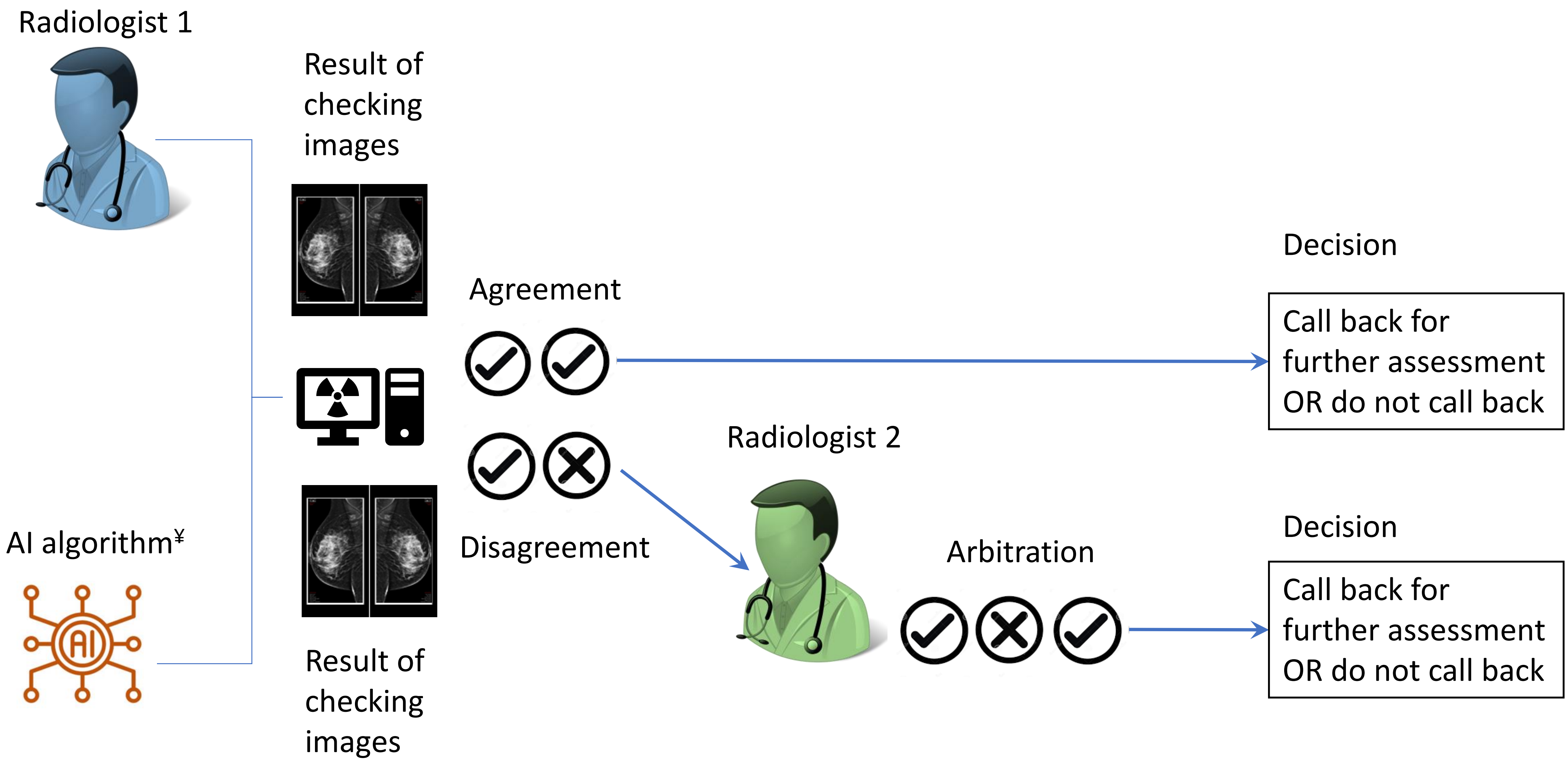

¥algorithm - set of rules or instructions to follow in the decision-making process

Scenario 2: AI would flag up for two readers (radiologists) which images they may want to look at first

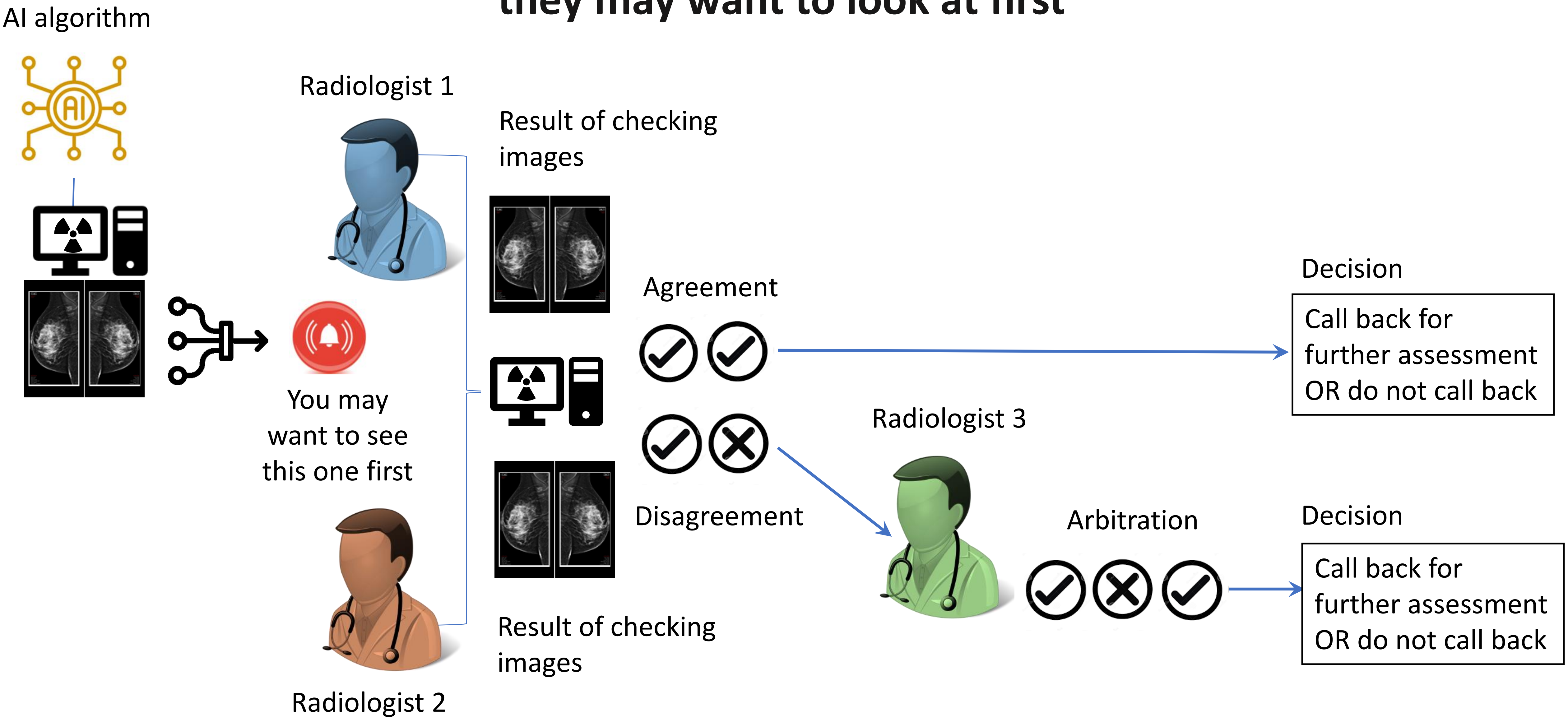

¥algorithm - set of rules or instructions to follow in the decision-making process
