# Supplementary material for: A stakeholder analysis to prepare for real-world evaluation of integrating artificial intelligent algorithms into breast screening (PREP-AIR study): a qualitative study using the WHO guide
Source: BMC Health Serv Res. 2024 May 2;24:569. doi: 10.1186/s12913-024-10926-z (PMC11067265; doi:10.1186/s12913-024-10926-z)
Supplement: Supplementary file 6 — Supplementary Material 6 [file 12913_2024_10926_MOESM6_ESM.docx]

Additional file 6: Description of strategies

| **Strategies** | **Objectives** | **Targeted stakeholders** (based on influence) | **Example quotes from our study participants** | **Considerations** |
| --- | --- | --- | --- | --- |
| 1. **Improve knowledge of AI for all stakeholders** | Convince stakeholders of the merits of the proposed reform as to how we articulate it would help shape the debate surrounding it. | High to low potential to influence (i.e., all ‘important’ stakeholders) | *… as long as I had the relevant information that I could interrogate and see and make a choice for myself as to what’s going to happen. (Focus Group 2)*  *Again just getting the word out there that it is a successful system that’s proven to work and could be used to help the workflow rather than hinder it in any way, so just getting the information out there. (ID 6)* | - Clarify the objectives and consequences of the reform using evidence from high-quality studies. - Coproduce and validate the information before distribution. - Make the information available: - In simple language (e.g., without technical/medical jargon), which should be understandable by lay individuals. - In related resources (hard copies, web links) via reliable sources such as NHS, cancer charities, and Science centres. - Use multiple activities with consistent messages, such as print (newspaper, magazines), podcasts, and broadcasts (television and radio). |
| 1. **Improve practical skills and ability of stakeholders directly involved in delivering the service** | Provide training to improve their ability to work with AI (e.g., to enable correct clinical decisions), which would improve their acceptance and support of the reform. | High to medium potential to influence  (i.e., important stakeholders such as readers, NSD) | *Well, the people must be taught how to use the new systems, so there’ll be an element of learning. (ID 20)*  *… At a bottom sort of level" to understand by everyone, professionals too. (ID 9)*  *… So actually …showing it in reality rather than just in words on a dry paper is sometimes useful. (ID 6)* | - Generate practical information based on their role and the policy and distribute it in the form of videos, and diagrams. - Identify training needs (via survey, discussion, or observation during a prospective study) and deliver via:      - Electronic module (e.g., video) - Demonstration by a colleague - User manual of specific AI system |
| 1. **Empower women to influence the screening service** | Empower service users such as women, the public, and patients’ representatives to communicate their knowledge and experience of the AI-assisted screening service to positively influence others’ trust and increase support for the reform. | Low potential to influence  (i.e., important stakeholders such as women and public and patients’ representatives) | *I think there’s another powerful message is you know, talking to people. So as an individual you have conversations with other women and whether it’s over a coffee or a game of golf or what have you, you can influence people that way. … So one person doing it using their own networks, their friends, … can be a very powerful influencer’. (Focus group 2)*  *I think … as an individual you have conversations with other women and … you can influence people that way. (Focus group 1)*  *think you’ll find very few people saying “No”, as long as you can assure them that it’s safe … But then the media including social doesn’t help because obviously, a bad news story is a much bigger seller than a good news story (ID 2)* | - Identify and recruit members of the community and target groups rather than individuals where possible, including: - Public and patients’ representatives (e.g., champions) - Women’s network of particular faiths, ethnicities, vulnerable groups - Public and patient involvement (PPI) group through industry and academia - Request their participation in defining, developing (using suitable language) and promoting local-level communication using case studies, and testimonies via - voice in radio - posters in screening centres - social media |
| 1. **Use a collaboration and team approach involving stakeholders to address challenges and improve support** | Engage relevant stakeholders (e.g., deployment-related) from the beginning of a reform process to increase its success and sustainability. | High to medium potential to influence  (i.e., important stakeholders such as NSD, hospital management group) | *I think probably a real collaborative and team approach. I think there’s an awful lot of people… and I think it’s about making sure that everybody is pulled in. … I suppose particularly if you get a site where you don’t have somebody who’s a real champion for AI it could be quite difficult. (ID 12)*  *…, it will be the set up and the digital infrastructure I suppose to ensure that all the partners join together. Because I think there will be a number of stakeholders involved with that. So it will be probably ensuring that we’ve got it set up properly and it’s all focused and in place (ID 4)* | - Discuss the proposed reform (e.g., use a tactic model and round table discussion) to - Identify barriers and facilitators towards the change process - Generate solutions for some common issues related to implementation and sustainability e.g., an AI guru with contact details. - Identify a champion/leader to communicate/convince, lead others to improve its support. |
| 1. **Improve real-world evaluations of AI** | Maintain and improve support by evidence synthesis and addressing stakeholders’ expectations of the policy. | High to low potential to influence (i.e., including all ‘important’ stakeholders) | *I think there need to be these really good clinical trials, we need to know it’s not missing anything that we normally bring back (ID 1)*  *I’d be... well, happy for it to be done if I had the evidence that it worked, that say it works successfully and safely, … (ID 19)*  *I would like to see proper studies that show that AI can actually work, can actually detect the disease as well as a human, … I think the second stage is actually what are the outcomes for patients and what are the outcomes for the service, because just being able to detect the disease doesn’t necessarily mean that as a screening programme it actually brings improvement. (ID 12)*  *So if there were randomised controlled trials, if we used it and if it was shown to just be correct all the time and not making any mistakes you would have to start to learn to trust it. (ID 13, moderate opponent)*  *And we don’t want AI to speed us to so fast that we can’t then offer an assessment appointment. So I think we’ve just got to watch the balance of where we’re going with it. (ID 4)*  *Then with my procurement hat on as well, I don’t think anybody has been able to give me any convincing figures around AI and actually what it’s going to cost and whether on a cost-benefit analysis it’s actually worthwhile. So I just feel that I’d like to see a bit more evidence on all that. (ID 1)*  *Upgrading AI software will cost. But if changed or upgraded, what are the impacts there and how do we get over that? (ID 18)* | - Design and conduct prospective studies for evidence synthesis in real world settings of NHSBSP - Compare AI’s performance to a standard or best/current practice - Studies should be conducted and reviewed by independent researchers (i.e., other than AI developers) to avoid any confusions and bias in findings - Evidence expected by our stakeholders is listed below: - Accuracy of detection e.g., whether accurate and reproducible - Safety in relation to the quality and consistency of AI’s performance - Feasibility for the purpose e.g., whether the policy is meeting its purpose - Impact on patient related outcomes e.g., anxiety - Social acceptance e.g., experiences and acceptance of the service users and providers - Operational/organisational effectiveness e.g., impact on the NHSBSP, Scottish National, Breast Screening Service and overall NHS - Economic impacts by conducting cost-utility, cost-consequence and/or cost-benefit analysis to capture whether the proposed reform is affordable and worth it. |
